# What makes a major change of wildlife management policy possible? Institutional analysis of Polish wolf governance

**DOI:** 10.1371/journal.pone.0231601

**Published:** 2020-04-23

**Authors:** Krzysztof Niedziałkowski, Renata Putkowska-Smoter

**Affiliations:** Institute of Philosophy and Sociology, Polish Academy of Sciences, Warsaw, Poland; University of Bucharest, ROMANIA

## Abstract

Poland was one of the first countries of Central and Eastern Europe with stable wolf populations to effectively introduce year-round protection of the species. This paper traces the process of policy change using institutional theory as an organizational perspective. Based on the analysis of data from desk research and semi-structured interviews, we propose a model of institutional change and argue that in the 1990s, environmental activists and wildlife biologists successfully used a political window of opportunity connected with socio-economic transformation after 1989 and managed to induce the government to move the species from the domain of hunting to the domain of nature conservation. The new policy, informed by an ecological paradigm, diverged from the historical path dominated by hunters and the vision of the wolf as a pest and a hunting target. The improved protection led to the numerical growth of Poland's wolves and ultimately to their westward expansion.

## Introduction

Wolf populations in Europe are on the rise [1, 2]. Among the reasons for this, Chapron et al. [2] and Boitani and Linnell [3] enumerate the changing of hunting practices, the introduction of European conservation legislation (Bern Convention of 1979, Habitats Directive of 1992), significant changes in public opinion in many countries regarding carnivore conservation, stable political institutions conducive to proper law enforcement, and the relatively smooth political transformation of the post-communist countries that facilitated sustainable forestry and hunting practices. They also suggest that the recovery of wolves and other carnivores have been aided by socio-economic changes that have decreased the pressure of human persecution (e.g. large scale rural-urban migration and land abandonment), improvement in habitat quality for wolves and their prey (e.g. through reforestation), the recovery of prey populations due to changes in hunting management, and the introduction of a variety of practices (such as livestock protection measures) that have improved coexistence between large carnivores and people. These accounts show that wolf recovery in Europe has been supported by a combination of socio-economic and biological factors. In the former case, however, natural scientists predominantly indicate that impersonal structural mechanisms have influenced the wolf come-back and rarely mention underlying socio-political factors connected with the values, beliefs, and activities of concrete social actors interested in wolf management.

At the same time, the literature points to social conflicts between groups with different interests and values towards wolves and other carnivores, especially in countries where the species has moved in relatively recently [4]. The key conflicts concern the depredation of wolves on livestock, their impact on game animals and hunting dogs (especially in northern Europe), and safety among some rural populations [5]. Furthermore, wolves have become proxies for many wider political and social tensions among urban versus rural communities, proponents of preservation versus proponents of using resources, and recreation-based economies versus extraction-based economies [6–9]. These tensions influence wolf governance in respective countries—a set of formal and informal rules (institutions) regulating who participates in decision-making concerning wolves, how the decision-making is organized, and what the wolf management purposes and instruments are [10]. Consequently, wolf management decision-making is as much a socio-political issue as a biological one [11]. So far, however, unlike in the United States [12], the recovery of wolves in Europe has attracted relatively little attention with regard to the socio-political factors underlying the introduction of legislation conducive to wolf recovery.

**Fig 1:**
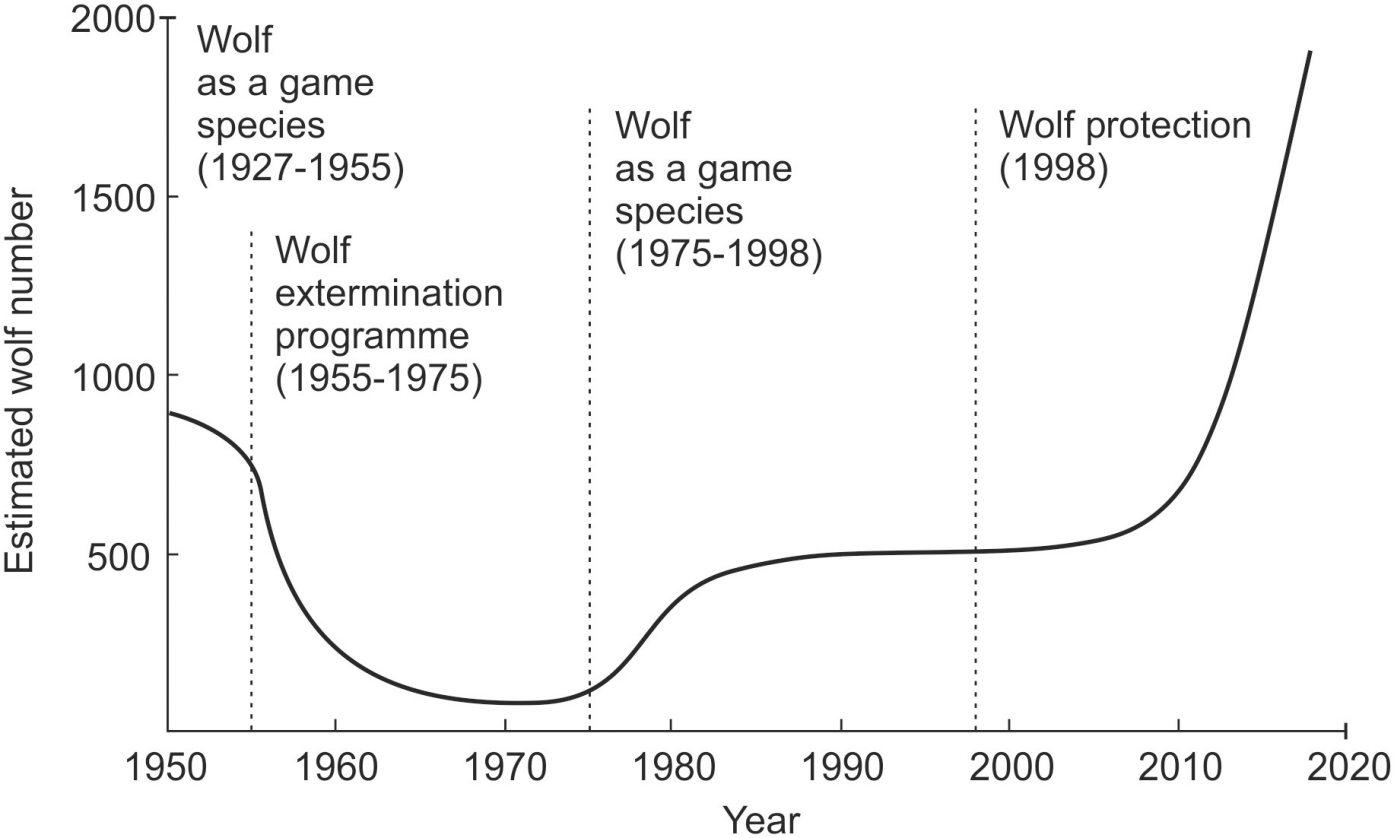
Estimated number of wolves in Poland and the legal status of the species (1950–2018). Source: 1950–1975: Sumiński [16] and Wolsan, Bieniek [17]; 1975–2018: Śmietana [18].

**Fig 2:**
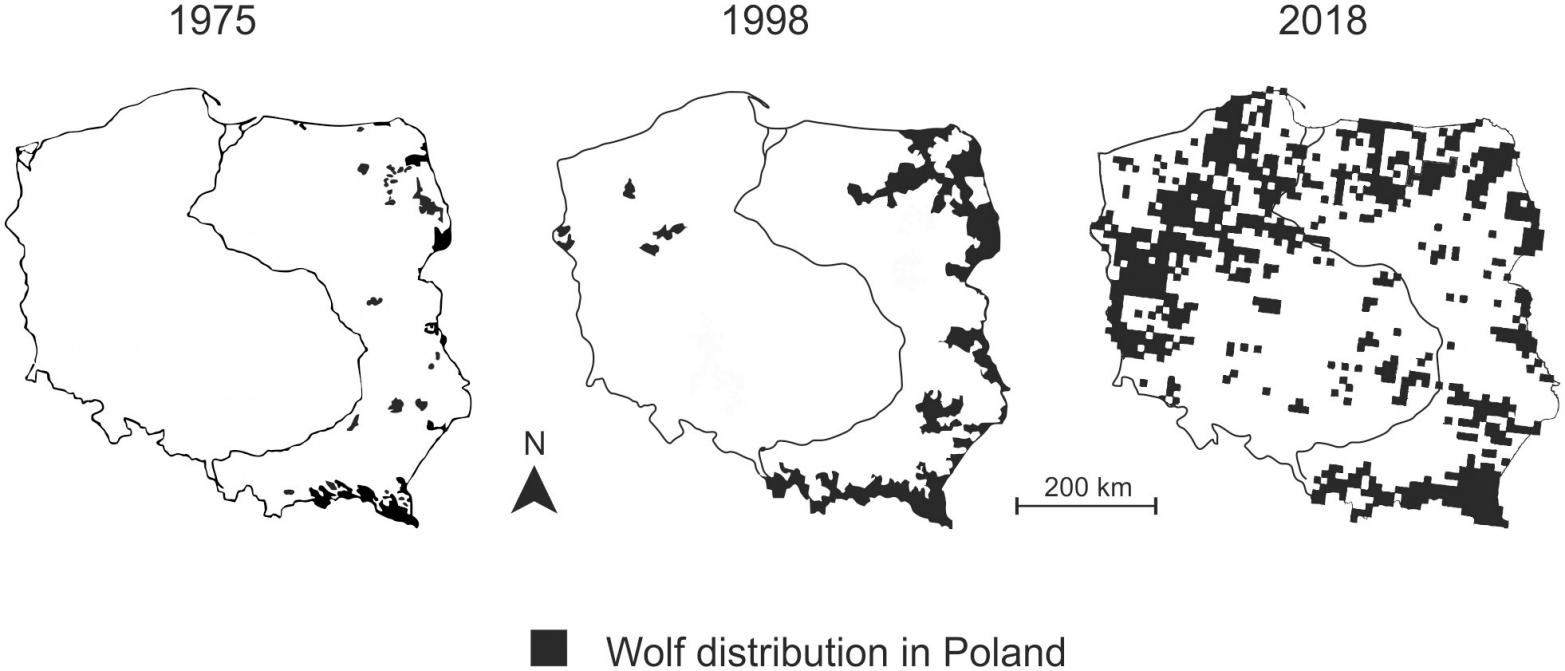
Wolf distribution in Poland in 1975, 1998, and 2018. Sources: Adapted from Wolsan, Bieniek [17]; Okarma, Jędrzejewski [19]; Śmietana [18].

This paper aims to provide an empirical account of the impact of the activities of social actors on the major changes of wolf governance at the national level by tracing the process leading to the enlistment of the wolf as strictly protected in Poland in 1998. Poland was the only country in Central and Eastern Europe that effectively banned wolf hunting before joining the EU (in 2004) and adopting the Habitat Directive. We aim to identify the conditions that facilitated the path-breaking legal and organizational changes ultimately leading to the growth of the Polish wolf populations (Fig 1) and their dispersal from eastern to western Poland (Fig 2) and then to Germany and other western European countries [1, 13]. Another goal of the paper is to propose a model of institutional change, informed by institutional theory [14, 15], that provides a conceptual background for understanding the mechanism of the transformation of wolf governance.

In the following sections, we first discuss the institutional approach to environmental governance and introduce a model of institutional change. We then describe methods and provide an account of the process leading to the protection of the wolf in Poland in 1998. The paper finishes with discussion of the results and conclusions.

### Environmental governance and institutions

Environmental problems can be interpreted as social phenomena where specific social groups find changing relations between society and nature problematic [20]. The interactions between environmental and social issues are “mediated through culture, economy and politics, and are constructed and re-constructed through conflict”[21, p. 173]. Public policies devised to deal with environmental problems are outcomes of socio-political interactions (from cooperation to conflict) between various actors with different values and resources and in a changing social, political, economic, and environmental context. Through these socio-political interactions, societal and policy goals are identified, trade-offs between competing objectives and resource allocations are established, and decisions are enforced [22]. These interactions influence their social context—the social structures and culture within which they take place—which, subsequently, has an impact on further interactions [23, 24]. The effectiveness of social actors in influencing policy process is determined by their power, which can be identified as “the organizational and discursive capacity of agencies (…) to achieve outcomes in social practices, a capacity which is however co-determined by the structural power of those social institutions in which these agencies are embedded” [25, p. 347]. In their interactions, social actors are informed by their discourse–“a specific ensemble of ideas, concepts, and categorizations that are produced, reproduced, and transformed in a particular set of practices and through which meaning is given to physical and social realities” [26, p. 44]. Knowledge of the world is constructed within certain discourses and is embedded in power relations [27]. In the context of public policies, discourse can be defined more specifically “in terms of its content, as a set of policy ideas and values, and in terms of its usage, as a process of interaction focused on policy formulation and communication” [28, p. 184].

The interactions of actors within “fields” or “arenas” [29] over time solidify into patterns—institutions—consisting of “cognitive, normative, and regulative structures and activities that provide stability and meaning to social behaviour” [30, p. 33]. Institutions constitute enduring elements in social life: they structure interactions and evolve gradually in response to those interactions, thus “connecting the past with the present and the future” [31, p. 97]. Institutions regarding environmental resources constitute environmental governance and temporarily resolve conflicts between groups of actors that represent competing interests and discourses regarding those resources [32]. Current institutions may be conceptualized as political legacies of historical struggles between competing groups. These legacies favour certain interests and discourses regarding reality, relevant knowledge, distribution of scarce environmental resources, and attempts from those groups to dominate their policy fields [33–35]. The ability of groups of actors to influence institutions is mediated by their “institutional capacity” [36], i.e. the power to sustain or disrupt existing institutions due to access to different forms of capital (intellectual, social, political, material). Through their more or less strategic actions regarding institutions [37, 38], actors want the state to explicitly or implicitly acknowledge their discourse in the form of environmental policies that correspondingly identify the nature of the environmental problems to be addressed, the ways of addressing them, and the people who should be involved [39, 40].

**Fig 3:**
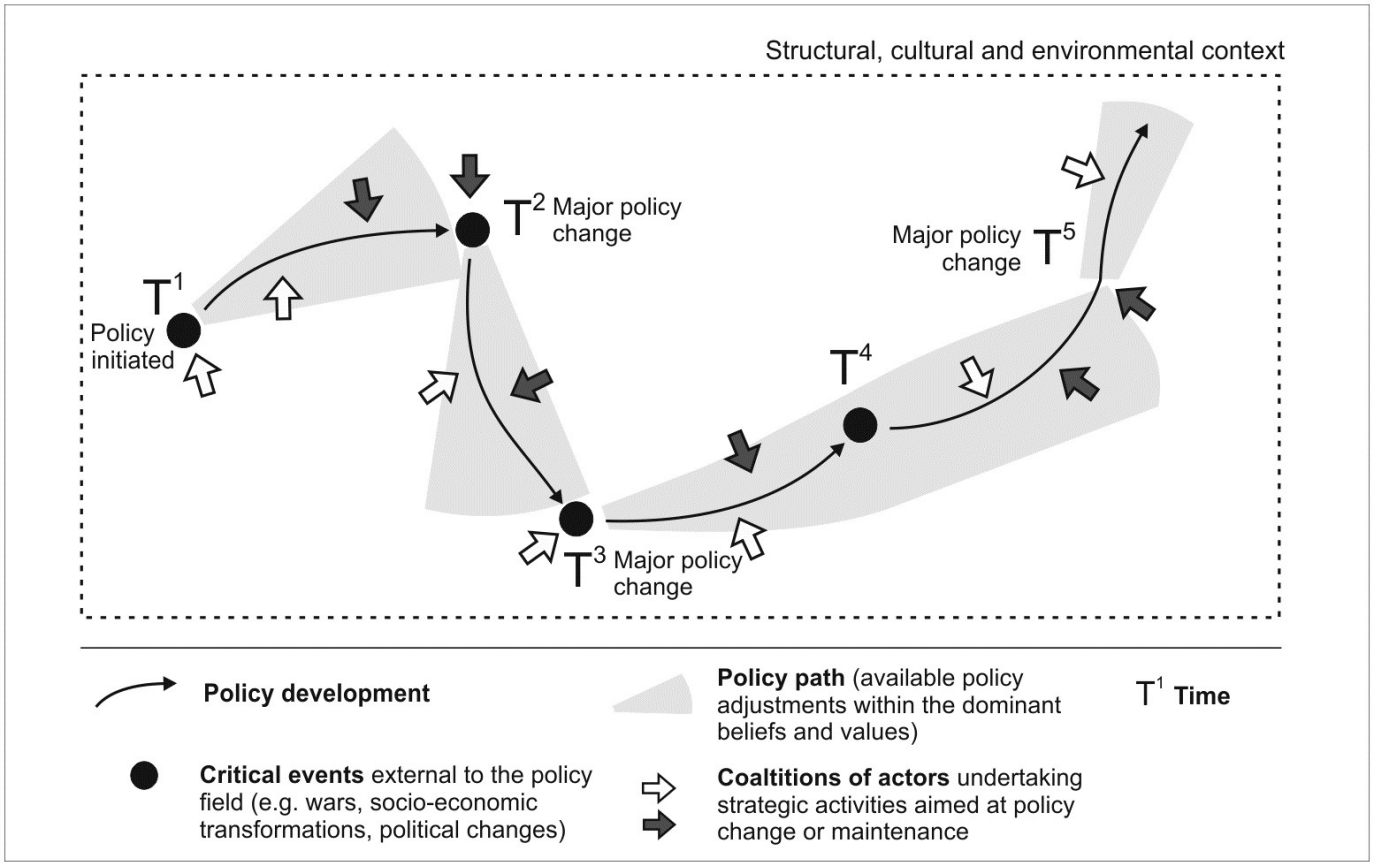
Model of policy change.

Institutional arrangements embodied in environmental governance are “path-dependant” and increasingly difficult to challenge over time as they become more reinforced, and alternative solutions become more and more difficult to implement [41]. Still, they evolve to fit the changing socio-economic, political, technological, and cultural context; to absorb new information; or to respond to the conflicts between actors involved in a particular policy field, especially conflicts over ideas and assumptions underlying policies [42–44]. These gradual adjustments and adaptations within ‘normal policy-making’ [39], including policy changes of the first order (routinized decision-making, settings of existing policy instruments) and of the second order (introduction of new policy instruments), might eventually amount to a major, paradigmatic, third-order policy change transforming the basic assumptions behind the policy [45]. However, usually, such a major transformation requires some external critical shock event that undermines the position of the dominant coalition of actors, destabilises existing institutional arrangements, and creates a window of opportunity for competing coalitions to shape the policy in line with their core beliefs [43, 46, 47]. Consequently, major policy change involves arguments that are more political than scientific and is accompanied by a change of groups deemed authoritative in the policy field [39]. Shock events might include political, economic, technological, and environmental “punctuations” as well as policy decisions and impacts from other policy fields [48]. Based on these premises concerning institutional change, we have developed a model of policy change (Fig 3). The model is informed by earlier frameworks for policy analysis, particularly Advocacy Coalition Framework [49] and Punctuated-Equilibrium Theory [50], as well as by institutional approaches to the analysis of environmental governance: Policy Arrangement Approach [51], and Evolutionary Governance Theory [35]. It is a heuristic and analytical tool that facilitates tracing the direction and dynamics of institutions over time.

Policy development (thin black arrow) is initiated by a group of social actors (white arrow) following a major event external to the policy field (black dot) at T^1^. During the formative moment, basic institutional parameters of policy (paradigms, rules, networks) are determined that then translate into a policy path. The shaded area of the policy path delineates normal policy-making—possible minor (first and second order) policy changes undertaken within a certain policy paradigm. These changes are stimulated by dominant actors in the field to improve the effectiveness of policies or to respond to criticism of challenging social actors who want to disrupt existing institutions (grey arrows). They might also be oriented at responding to changes in surrounding fields and/or in a wider structural, cultural, or environmental context of policy-making. Usually, policy development experiences a major (third-order) change following some critical external disruptions (black dots—wars, major socio-economic transformations and political changes, environmental disasters, etc.). These disruptions influence the institutional capacity of policy actors. At T^2^, the challenging group of policy actors supporting a new paradigm manages to transform the direction of policy development to follow a new path informed by a new policy paradigm, which will last until the next major change initiated by a punctuation at T^3^. Major external events do not always amount to third-order policy changes, and institutional arrangements might prove resilient to the stress (as in T^4^). It is also possible that a series of first and second-order changes may induce a major policy change (at T^5^), even in the absence of critical external events.

Following the theoretical insights described above, wolf governance might be conceptualised as a field of public policy comprised of institutions regulating the goals and methods of wolf management as well as groups of actors involved. This public policy is informed by a certain dominant way of interpreting wolves and the need for their management. The dynamics of policy involves changes of different degrees that are influenced by the relations between groups interested in wolf management and by the wider socio-political, economic, and environmental factors that impacts those relations. In the following sections, we use the model above to interpret the changes in wolf policy in Poland starting from 1945 and finishing with the listing of the species as protected in 1998.

## Methods

To reconstruct the process of change in wolf policy, we carried out desk research and identified 226 texts concerning wolf management published between 1953 and 2019, including scientific papers, articles in the main specialist periodicals (hunting, forestry, nature conservation), articles in mainstream newspapers and magazines, and leaflets. A substantial part of the analysed collection constituted legal regulations, parliamentary proceedings, strategies, and reports on official and formal directions of wolf policy. Most of the identified texts were published in the 1990s and address wolf management in this period. In our view, this corpus covers practically all relevant sources concerning the analysed issue both from the environmental and the hunting side. Additionally, to triangulate the written data, we conducted 12 semi-structured open-ended interviews either with people involved in the campaign for wolf protection (identified based on written sources) or other actors who could shed some light on the introduction of protection and its circumstances (based on their experience and profession). Our purposive sample included two NGO members, seven scientists (specializing in wildlife ecology, hunting management, and forestry), two hunting foresters, and a former public official. We recruited new interviewees until new interviews provided relatively little new information on the process of policy change in 1998. All potential interviewees but one (a scientist) agreed to be interviewed and provided informed verbal consent recorded at the beginning of the interview. Interviews, assisted by an interview guide (S1 Appendix), were carried out by the authors of the paper and lasted between 0.5 and 2 hours. They were recorded, anonymized, and transcribed. We used Atlas.ti software to code and analyse the collected data. The analysis followed directed content analysis [52] and focused on formal and informal “rules of the game” within the field of wolf policy, important actors and the relations between them, their discourses on wolf management and nature conservation in general, and their capacities to influence policy-making.

### Wolf governance in Poland (1945–1998)

After the Second World War, wolf populations in Poland increased both in range and size, reaching around 800 individuals in 1951 and inhabiting major forested areas in eastern Poland and the Polish Carpathians [17, 53]. The species was legally classified as a game animal that could be hunted throughout the year. Hunters were interested in the highest possible reduction of its populations to limit losses caused by wolf depredation on wild ungulates and to remove alleged threats to humans [54]. Following accounts of the increasing predation of wolves on farm animals and wildlife, in 1955, the national government excluded the wolf from the list of game species and proclaimed a programme for the extirpation of the ‘pest’ [55]. “Akcja wilcza” [eng. Wolf action], allowed for a wide range of methods (including poisoning) for killing wolves to be used and offered high bounties for killed animals (ibid.). In 1959, the population was assessed at 250 individuals [56] (see also Fig 1), but the programme continued until 1975 when the wolf was relisted as a game species, bounties were cancelled, and a close season from 1 April to 31 July was introduced (apart from the main wolf areas where seasonal protection was instituted in 1981). At that time, there were less than 60 wolves in Poland, mainly in the eastern-most parts of the country [16]. This relisting of the wolf as a game species was spurred by the warnings of scientists that the species might be exterminated and by the growing awareness of the potential positive impact of wolves on ecosystems [54, 55]. However, the lobbying of groups supporting wolf eradication contributed to the reintroduction of high bounties in 1984 (without cancelling of the game status). This was met with opposition from environmental activists and wildlife biologists, whose pressure led to the final cancelling of bounties in 1989 [57]. In 1975, wolf numbers started growing and, according to hunting statistics, they reached almost 900 by 1990 [58].

The wolf governance under the hunting legislation was dominated by hunters associated with the Polish Hunting Association and foresters, many of whom were hunters themselves. Wolf policy was supervised by the Ministry of Forestry and administered by public officials responsible for hunting. The whole country was divided into hunting districts managed usually either by hunting clubs (under the Polish Hunting Association) or by the State Forest Administration. Hunting was regulated by annual hunting plans prepared by the entities managing particular hunting districts. Plans concerning forested areas had to be agreed with the forest administration. In some areas (e.g. Bielsko-Biała province, South Poland), hunters and foresters organized annual meetings during which they specified the number of wolves to be hunted in particular hunting districts [59]. Scientific advice was provided by specialists in hunting management.

During communist times, hunting was perceived mainly in terms of “hunting economy”, which was based on the breeding of game to be sold in order to support the national economy [60]. This had its effect on the perception of wolves by hunters. They viewed the species first and foremost as competition preying on the game animals that hunters were breeding. At the same time, they considered the wolf as a prestigious prey: the ultimate hunting achievement with a valuable trophy. In the hunters’ view, hunting helped to keep wolf populations at a ‘reasonable’ level and was necessary to prevent a proliferation of wolves that could threaten other game species (from an interview with a hunter). They also viewed hunting as a way to limit the predation of wolves on livestock. Wolves dispersing to the western part of Poland were eliminated so that the wolf populations were practically restricted to forest pockets in eastern Poland.

In 1989, the political and socio-economic transformation of Poland started. It involved democratisation, new parties in power, transitioning from a centrally-planned to market economy, decentralisation, as well as opening the way for the activities of NGOs. In the early 1990s, NGOs enjoyed strong social legitimacy based on their role in the protest movements in the 1980s [61]. Due to international contacts and support programmes, their capacity was growing, and they professionalized and increasingly used expert support [62]. Environmental issues were also relatively high on the political agenda because of their perceived high role in the process of European integration [61]. These socio-political changes influenced the field of wolf governance.

Already in 1989, wolf hunting was prohibited in the Białowieża Forest (East Poland) due to ecological research concerning wolves carried out by wildlife biologists [55, 63]. One of the biologists involved explained in an interview: “In 1989, after political changes in Poland, our academic tutor [name] became vice-minister of environmental protection (…). We informed him that the numbers of wolves [provided by hunters] were overestimated and that hunting wolves in the Białowieża Forest affected wolf families that lived in the Białowieża National Park. Being a biologist and ecologist, he quickly understood the problem and introduced (…) protection for wolves in the Białowieża Forest”. Wolf research in the Białowieża Forest carried out by wildlife biologists with the novel technology of telemetry brought new data on wolf biology and ecology, which challenged some assumptions underlying wolf management by hunters (e.g. the number of animals) [e.g. 64]. Scientists criticised the hunting approach to wolves, arguing that it was “influenced by ideas from the 19^th^ century”, and condemned the excessive hunting period, the plans to kill all wolves in particular hunting districts, and the lack of responsibility for exceeding wolf hunting plans [65]. Instead, they argued that “scientific research has proven that large predators are indispensable elements of healthy ecosystems” and that “one of the goals of hunting policy should be the full protection of wolves” (ibid, p. 21).

The new socio-political context in Poland brought about legislative changes. In 1991, a new Nature Conservation Act was introduced. It stipulated that regional representatives of the government (voivoda—governor) could introduce the legal protection of fauna and flora species within their regions. In Poznań province (West Poland), a few wildlife biologists used this opportunity and in 1992, forwarded an official notion to the regional governor arguing that due to the hunting pressure, wolves cannot repopulate in the province [57]. Their notion was accepted, and they sought similar measures in the neighbouring provinces of Gorzów and Piła ‘despite some opposition of hunters’ [66]. In the same year, leading Polish zoologists published the ‘Polish Red Data Book of Animals’ [67], the first comprehensive publication of that sort in Poland, which listed the wolf under the category of ‘rare species’–‘represented by small, isolated and dispersed populations with a high risk of extinction’–and suggested increasing protection measures.

These events challenged the perception underlying wolf policy that the condition of the species under hunting management was favourable. They also invigorated a campaign for the full protection of the wolf and the lynx initiated in 1991 by a newly established environmental NGO “Workshop for All Beings” in Bielsko-Biała province. By the end of 1993, the activists managed to convince the regional governor to introduce practically full protection of the species and expanded their campaign to the other parts of Poland. They collaborated with leading wildlife biologists specializing in wolf ecology and prepared educational materials with arguments supporting wolf conservation and highlighting wolves’ positive role in ecosystems. The NGO’s strategies included gathering signatures for petitions to protect the wolf and sending these to the government and to the regional governors; encouraging sending personal letters to decision-makers; producing and distributing leaflets; organising marches, lectures, and music concerts; contacting the media; and personally meeting decision-makers [68]. The latter strategy was facilitated by the changes of relations between decision-makers and activists following the greater accessibility of politicians since 1989. As recollected by the interviewed activists: “At that time, you could enter the office in the Ministry and start talking to the minister, and now you need to pass four thousand security gates”; “It was quite easy for me to meet the minister. I had a stack of petitions and I just barged into the office. I tried not to negotiate with lower level officials because I knew that it was a hierarchical organisation”. These meetings were also aimed at helping the officials to introduce changes advocated by activists: “The officials were writing justifications for legal regulations and this [required] providing them with arguments. (…) Sometimes you needed to give them a piece of paper [with arguments] so that they could use them”.

However, in the activists’ view, the government itself did not see the need to protect the wolf. This may have in some part been because some of its members were hunters, whom activists perceived as their main opponents. Only the bottom-up societal pressure showing that “in the young emerging democracy there was a social need to protect large carnivores” could, in their view, bring positive results (Interview with NGO). The political and social circumstances in the early 1990s proved conducive to such actions: “The authorities were more susceptible to the voices of social actors. (…) Practically, the first protests in Poland [after democratic transition] concerned the [protection of] Białowieża Forest and large carnivores. No one was used to that and it was a juicy piece for the media. No one was used to [a situation] where people stand on the street and demand something that is not connected with bread, meat, sugar, etc.“. The activists were particularly focused on contact with the media: “Media were already free and could cover what they wanted. They were very interested [in our activities]. Numerous articles on this subject were published in various newspapers and magazines (…) We used to have a strategy to inform the media. (…) We sent [information about] everything that was going on in the campaign (…) to the press (…) so that they knew what was going on, that there was a need, that society demanded…“. Wolf conservation demands were published in mainstream national media which, according to activists, was particularly important for pressing regional politicians. At that time, Poland was still divided into 49 small provinces (in 1998 this went down to 16): “Environmental activists in mainstream national media were some kind of signal for the local governors—these provinces were minute; these were some second-rate officials (…) who felt pressure from above” (Interview with NGO).

The campaign by environmental activists was accompanied by discussion about the need for wolf protection among scientists interested in wolf management. Two positions could be distinguished. Most wildlife biologists, including the State Committee for Nature Conservation (an advisory body to the minister of environment) treated the wolf as an endangered species requiring higher conservation measures [55, 69–72]. They agreed that in contrast to the traditional image of ‘the big bad wolf’, which had informed wolf policy in Poland and led to wolf persecution, the wolf played an important ecological role in the ecosystems, especially with regard to ungulates. Because of that, the species should be more widely distributed. They suggested that official statistics concerning wolves might have been overestimated and the hunting pressure too significant for the species’ restitution. At the same time, they indicated that international regulations that concerned Poland (CITES) or were about to concern (the Bern Convention) treated the wolf as endangered. In their view, Poland should follow suit and adjust the legal rules of wolf management to restore the population. Another group of scientists, specializing in hunting management, opposed changing wolf policy [73, 74]. They argued that hunting had not limited the increase of wolf populations and should be continued without new conservation measures. They also disputed the presumption that official wolf numbers were overestimated. In their opinion, the campaign to protect the wolf was not based on reliable data. Before changing policy, decision-makers should listen to the ‘opposing side: hunters, foresters, and scientists’. According to those hunting specialists, increased protection of wolves would worsen their situation because animals would be killed illegally, while hunters would not be interested in intervening. Finally, the protected wolf might lose its fear towards humans and increasingly attack livestock. Consequently, they recommended keeping the status of the wolf as game.

While the position of hunters towards wolf protection was negative, the approach of foresters was more nuanced. On one hand, many foresters were hunters themselves and subscribed to their values and beliefs concerning wolves. Furthermore, many hunting districts (e.g. in Krosno province) were managed by forestry units that benefitted from organising wolf hunts for Polish and international hunters. On the other hand, to limit damages in forest plantations, foresters were keen on reducing the densities of wild ungulates kept at high levels for hunting purposes before 1989 [75]. Environmental activists tried to use these arguments to win foresters’ support: ‘We were arguing that the wolf is needed in the forest, that it is an ally of forest management’. Despite these attempts, at the local and regional levels, foresters did not particularly endorse wolf protection, and sometimes outright criticized conservation initiatives [76]. At the national level, such arguments proved more effective, especially because environmental activists were strategically using evidence provided by scientists specialising in forest management: ‘We were providing the Ministry with arguments for wolf protection, showing that wolves play an important ecological role in limiting wild ungulates based on evidence published by forestry scholars. And because the Ministry of Environment is very forestry-oriented and always has been (…), these arguments were more effective than those presenting wolves as beautiful or rare” (Interview with NGO).

As a result of the campaign of activists and some wildlife biologists, by the end of 1994, several of the 49 provinces in Poland introduced wolf protection or strongly reduced the hunting season. Activists also criticised wolf hunting plans in other provinces and started mobilising international support—for instance, in January 1995, Brigitte Bardot wrote a letter to president Lech Wałęsa to stop planned wolf hunts in Krosno province. In March 1995, the government signed the Bern Convention (ratified on September that year), but with a reservation that in Poland the wolf would not be “strictly protected”, as declared in Appendix II [77]. One month later, In April 1995, the government issued a new regulation concerning protected wildlife species and designated the wolf as protected in all but three provinces with particularly high wolf densities—Krosno, Suwałki and Przemyśl. In the two latter provinces, however, wolves became protected following decisions from the regional governors, so hunting was allowed practically only in Krosno province. It hosted the biggest wolf population at that time, which was considered by some wildlife biologists as particularly valuable for the sustainability of wolves in Poland [66]. Environmental activists, supported by scientific data provided by a wildlife biologist investigating wolf ecology in Krosno province, tried to convince the regional governor to list the species as protected regionally, however, strong criticism, especially from hunters, foresters, herders, and contrary evidence from a specialist in hunting management, led to the eventual rejection of the campaign [68, 73]. At the same time, the Polish Hunting Association was trying to convince the Ministry of Environment to restore wolf hunting in several provinces that hosted around 90% of the Polish wolf population [78].

The pressure of the activists to list the wolf as protected in Krosno province continued. In the meantime, some leading activists from the Workshop for All Beings established a new NGO called “Association for Nature ‘Wolf’”, which focused in particular on wolf conservation. Activists tried to convince the regional governor in Krosno to limit the number of wolves to be hunted and the minister to introduce the protection of the wolf in the whole country. According to the interviewed members of NGOs, the latter was hindered by the then-minister of environment Stanisław Żelichowski (1993–1997): “Żelichowski was a hunter (…). We had a few skirmishes concerning the wolf. (…) The attempts to introduce protection in all provinces seemed hopeless because he was firmly against it”.

Parliamentary elections in 1997 and the establishment of a new government, including the new minister of environment Jan Szyszko, created a new political situation, which in the view of the interviewed NGO members proved crucial for the final change of legal rules: ‘When he was only about four days in office, I phoned him (…), and [knowing that he was a forester and was sensitive to forestry issues,] I told him that I would like to meet him and show him the result [of research] suggesting that the wolf was a great support for forest management (…). Szyszko wanted to show that [as a new minister,] he was open to NGO voices and invited us to give a talk for his students (…). He also invited journalists. (…) After the talk, the journalists attacked him for what he was going to do considering all the arguments [supporting wolf conservation], and (…) he blurted out that (…) everything suggested that the wolf should be protected. This was in February, and in April, the regulation [for protecting the wolf throughout the whole country] was issued’. The official justification for the regulation stated that ‘the wolf plays an important role in maintaining the ecological balance in the environment and is a natural regulator of the population number and health status of game ungulates’ [79, p. 125–126]. To address concerns at the local level, the new law allowed for intervention culls, e.g. to eliminate individuals posing a direct threat to humans. Introducing wolf protection was accompanied by other actions from environmental activists and wildlife biologists, who were aware that the legal protection could be removed or limited due to pressure from hunters or livestock owners. For instance, due to lobbying of a pro-wolf coalition, from 1997, the State Treasury was legally obliged to compensate for wolf damages to livestock. In parallel, activists provided free training and support to livestock owners to protect their herds against wolf attacks.

The listing of the wolf as a protected species under the Nature Conservation Act changed the composition of groups involved in wolf management. The administration of wolf conservation was transferred to provincial conservation offices at a regional level and to the chief nature conservator at the Ministry of Environment at the national one [75]. In 1998, the Ministry of Environment commissioned the preparation of a strategy of wolf management. It was co-authored by some of the most vocal wildlife biologists supporting conservation and the activist who had led the campaign [19]. The strategy set out to retain wolf conservation in Poland and proposed activities facilitating population increase in major forest areas in the country, mitigating damages in livestock, adjusting hunting practices and plans to consider wolves’ impact on game, and research, monitoring, and educational activities. Although never officially accepted by the Ministry due to the opposition of actors supporting wolf hunting, it initiated further activities from conservation actors who wanted to ensure the stability of the new arrangement. The protection of wolves became the cornerstone of wolf policy in Poland in the following years, additionally strengthened by the accession of Poland to the EU in 2004 and the adoption of the Habitat Directive.

## Discussion

**Fig 4:**
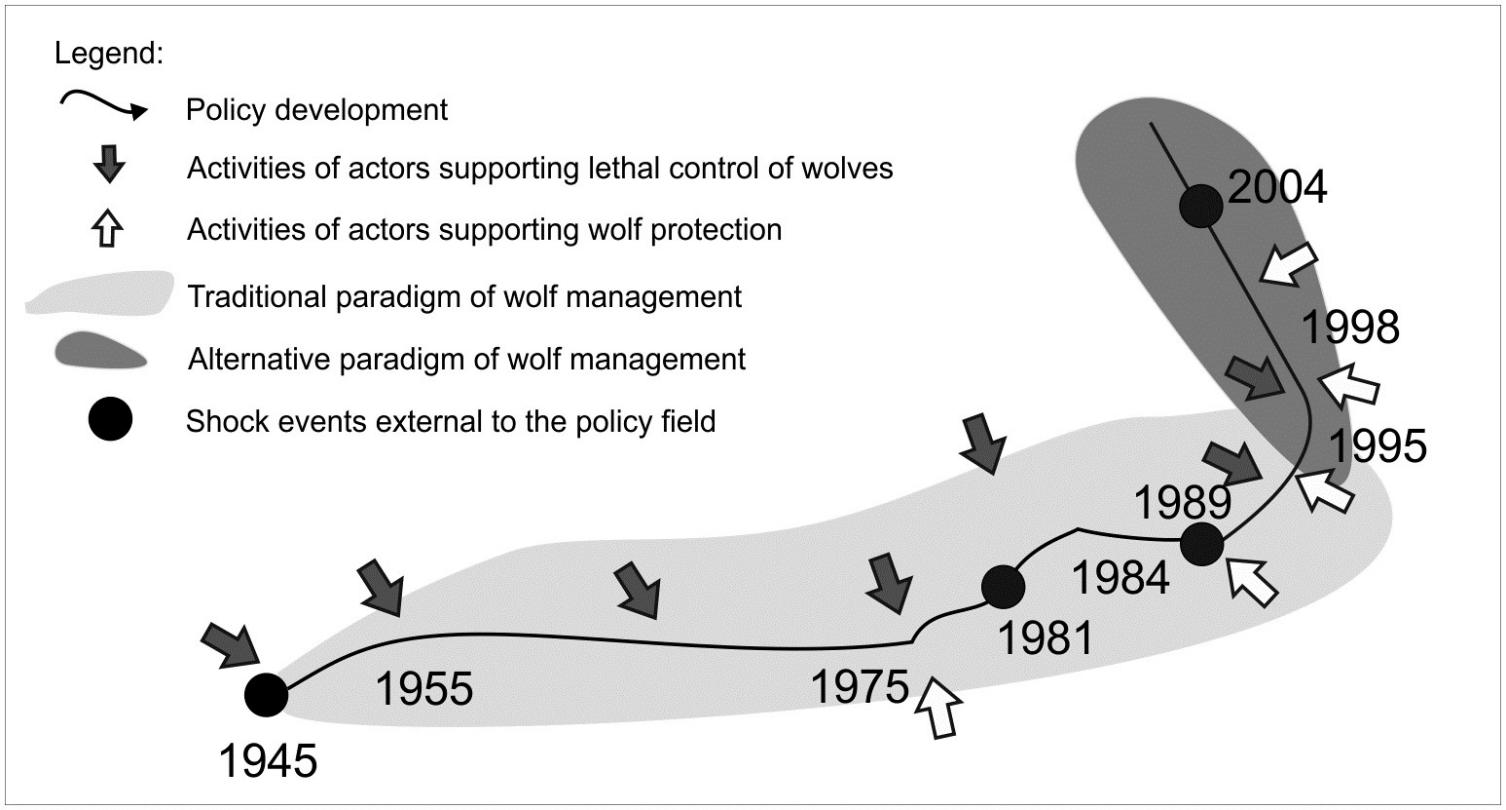
Institutional development of wolf policy in Poland (1945–2004).

Wolf governance in Poland can be interpreted as a field of public policy that includes a coherent set of institutions regulating wolf management and people involved. This field is guided by a particular paradigm concerning the perception of the wolf and its role in the environment. In the analysed period between 1945 and 1998, wolf policy underwent a major institutional change (Fig 4).

In 1945–1989, hunters and foresters dominated wolf governance in Poland. They perceived the wolf on one hand negatively as a dangerous and cunning predator threatening game, livestock, and humans, and, on the other hand, as an attractive hunting trophy. The policy focused on keeping the wolf population under strong human control. The perception of exceptionally high wolf numbers in the 1950s contributed to the introduction of new policy instruments—the programme of wolf extermination (1955–1975). However, it did not change the dominant policy path or the main groups of actors involved. After returning to the status of game animal in 1975 and 1981, a few minor policy changes followed that were connected with the cancellation and reintroduction of bounties. In 1989, a socio-political and economic transition started and significantly transformed the context of wolf governance. New actors (NGOs) could ally with established actors (wildlife biologists) with alternative discourses in the field (wolves as regulators of ecosystems in need of protection), and using new legal opportunities, resources, and strategies, influence policy-makers at regional and national levels. This resulted in a series of major policy changes at local levels transferring wolf policy from the domain of hunting to the domain of nature conservation and a major policy change at the national level in 1998, when wolves became protected throughout the whole country. The new path of wolf policy was informed by a new paradigm, according to which wolves play a positive role in ecosystems and their numbers should not be regulated to encourage their dispersal and allow for a natural recovery of the population. The new authoritative group in the field became wildlife biologists and NGOs.

The analysed changes in wolf policy follow a “punctuated equilibrium” scenario of institutional change, when periods of relative stability are punctuated by short periods of dramatic changes triggered by external events [46, 80]. The stability of the institutional path between 1945 and 1989 was connected with the dominant and, for a long period, practically unchallenged discourse on wolves as a problematic species that required constant lethal control and with the prevailing position of hunters and foresters in the field. Alternative discourse, creating the first building blocks of a new paradigm, started appearing when the wolf was close to extinction and contributed to the cancellation of the extermination programme and then to the cancellation of bounties. However, it took several years for the new discourse to mature, supported by technological advances in research methodology, and the external shock of 1989 for the institutional capacity of environmental actors to increase enough to challenge the dominance of hunters and foresters and to institutionalise the new paradigm. Our results point to the central position of ideas in the mechanism of institutional change [81, 82]. These ideas needed to be produced (e.g. the concept of the benefits of wolf predations for the ecosystem suggested by wildlife biologists) to constitute seeds of change in the direction of new institutions. In wolf conflicts, environmental actors usually take advantage of scientific discourse that is frequently privileged over other social discourses [8, 83]. In Poland, both groups of actors invoked scientific arguments and were supported by distinct epistemic communities, which, as brought up by Skogen, Mauz [84], displayed a different understanding of nature—either more environmental (wildlife ecologists) or more utilitarian (hunting biologists). These epistemic communities were also identified in other conflicts regarding wildlife management in Poland [85]. Scientists belonging to these communities were not only developing scientific discourse on wolves, but also directly engaged in pressing for institutional change (e.g. in Białowieża and in Poznań province) or in resisting it (as in Krosno province).

The ideas behind policy paradigms need to be promoted by actors with adequate resources and with access to policy-makers (in this case, NGOs). This highlights the importance of agency for major institutional change in wolf governance, which is a relative new finding in terms of wolf conservation in Europe. This is usually associated with top-down implementation of EU conservation rules and disempowering arrangements [10, 86]. However, structural factors also played their part in the wolf case. The socio-political transformation of Poland from state socialism to a liberal democracy and market economy constituted a key external factor: (1) it opened up possibilities for new groups of actors in the field (e.g. access to policy-makers and media); (2) it broke the *status quo* in the main policy arena for wolf policy at the governmental level and introduced new policy areas at regional levels; (3) it disturbed neighbouring politically and economically dominant policy fields of hunting and forestry; (4) it facilitated the transfer of environmental discourses and rules. Other wider social mechanisms that influenced wolf policies in Western Europe and in North America might have also played an indirect role. This concerns for example shifts in the share of urban population, which tend to have higher acceptance for wolves [87], from 37% in 1950 to 62% in 1990, and the general increase in concern about the state of the environment, which influenced attitudes towards nature [88, 89]. Unlike in the developed world, where environmental concerns expressed since the 1960 translated into a quick growth of the environmental movement and implementation of the new environmental legislation, in Poland they found its effect mainly after the collapse of the communist regime. Favourable conditions in the decade after the democratisation contributed to the heyday of environmental NGOs (particularly those centred on nature protection), adoption of new laws regulating nature conservation and environmental protection, and the proliferation of the initiatives aimed at the protection of natural areas and endangered species, including the wolf [90, 91]. Socio-political transition in the early 1990s also contributed to the legal protection of wolves in Slovenia [92] and Romania [93]. In Slovakia such attempts were undertaken in 1995–1999, but they were successfully opposed by hunters [94]. In the Baltic states, however, the socio-political changes did not lead to challenging of the status of the wolf as game [95].

This suggests that while the democratic transition might have opened the window of opportunity for a policy change by influencing structural characteristics of the wolf policy field and its wider context as well as by impacting on constraints and opportunities for policy actors, it was up to the activities of those actors to use these opportunities and, in the analysed case, they did. The chief nature conservator (1997–2001) stated during Parliamentary proceedings (25–26.10.2000) that ‘it was a tiny NGO called “Wolf” that induced the government to fully protect all wolves in Poland despite some contrary opinions from professional circles’. Our case suggests that, although the policy change was facilitated by a wider group of actors, wolf conservation was indeed brought about by a few policy entrepreneurs [96], who skilfully used the window of opportunity created by the changing socio-political conditions. To trigger institutional change, they engaged in various forms of institutional work [37] and contributed to establishing rules constraining their adversaries.

The analysed case of wolf policy revealed relatively weak opposition from the dominant actors in the field, who failed to protect their position and the prevailing paradigm of the wolf policy. The data suggests a few main reasons for this. Firstly, organisational changes within the fields of hunting (the new Hunting Act of 1995) and forestry (the new Forestry Act of 1991 and the potential threat of privatising public forests) focused the attention of these key groups on their key domains [97]. Secondly, hunters and foresters did not expect the policy change due to their dominant position and prevailing resources, and the perceived limited influence of the new actors in the field. As argued by Richardson [98], established actors facing a “virus” of new discourses can either change themselves, transform the “virus”, or accept losing their position in the field. In this case, in the face of ideational changes concerning wolf protection, hunters did not display flexibility and hardly changed their discourse or practices regarding wolf management. Thirdly, the dominant groups were bypassed by the challenging actors who lobbied policymakers directly, often without the dominant groups being aware of their actions. Many representatives of the previously dominant groups in the field of wolf governance were oblivious to the key factors and people contributing to the change, e.g. they suggested a letter from Brigitte Bardot to the President of Poland was a crucial cause of wolf conservation. Finally, the opposition to the new rules might have been limited by the lack of direct coexistence with the wolf among the large section of the hunting community and local communities due to very limited range of the species at that time [4, 99].

The way the major policy change was implemented had a top-down character and did not involve consultations with interested groups. It was far from participatory governance and deliberative processes advocated by the current literature on carnivore management and conservation conflicts [100, 101]. The literature on wolf governance particularly highlights the role of local communities in wolf conflicts and the need to negotiate policy responses with those communities to increase the legitimacy and effectiveness of new instruments [102, 103]. This, however, often proves challenging and the outcomes regarding trust building and conflict mitigation are very limited [104, 105]. In our case, local communities from existing or potential wolf territories did not participate in the policy-making, although some groups of local actors (especially livestock farmers) were particularly affected by the wolf’s presence. This can be explained by the dominance of top-down mechanisms of environmental governance in post-socialist states [106, 107]. Both at the national and regional levels, decisions concerning wolf conservation were in the end made respectively by the government or its regional representatives (governors) accountable to their superiors at the central level. Affected farmers lived in more remote parts of the country and did not have adequate resources to influence policy-making. Still, their concerns were considered by the groups competing in the field, although differently. While for hunters, damages caused by wolves to livestock showed a need for higher hunting quotas, for environmentalists, such damage required compensation schemes and support to farmers in protecting herds against the attacks. When policy change took place, environmental activists identified such interventions as crucial for the effective conservation of wolves in the future [108]. Despite the top-down mode of governance, the fact that decision-making after 1989 was increasingly influenced by representatives of civil society (NGOs) should be interpreted as a step toward democratisation of the process.

The final result of the conflict suggests a “win-lose” outcome [101], in which one side of the conflict asserts its views with little discussion with other parties, a solution is imposed and at least one party is effectively silenced. Such situations often result from fundamental differences in values of the conflicting parties and the perception among one of them that their interests will be better served by legislation and enforcement, rather than dialogue (ibid.). Following our model of policy change, we suggest that such “win-lose” scenarios are likely when the party whose values are not in line with the dominant policy paradigm perceives the change of this paradigm as possible, considering its institutional capacity and the political situation in the field. Participatory deliberative approaches are more likely when major change is not in view. These approaches would produce changes of first and second order that do not undermine the dominant policy paradigm but mitigate conservation conflicts and offer relative “win-win” solutions. Our case shows that “win-lose” scenarios do not necessarily lead to more conflicting in the long run than “win-win” solutions, as deliberative approaches suggest. It implies that the latter can be taken up to sustain the former. In Poland, introduction of the protection of wolves decreased the level of conflict because the protests of NGOs stopped, while hunters and livestock farmers did not challenge new rules considerably, although they tried to change them through lobbying among the policymakers [109]. The NGOs and policy-makers have engaged in facilitating “win-win” solutions to decrease conflict and prevent a potential major policy change—restoration of wolf hunting. Such activities may at some point require introduction of more collaborative approaches to support coexistence between humans and large carnivores in human-dominated landscapes [110]. As argued by Hartel et al. [111], collaborative efforts should be strengthened by transdisciplinary research and active engagement of researchers in creating new institutional structures. Our case confirms that, in a favourable context, such collaboration between stakeholder groups and academia can have a significant impact on institutional dynamics.

## Conclusions

The paper presented the process of changing wolf governance in Poland between 1945 and 1998 and interpreted it as an institutional phenomenon. Using a model of policy change, we argued that socio-political changes initiated in 1989 created a structural window of opportunity for environmental actors to critically change the dominant policy path based on the utilitarian perception of the wolf to a new path informed by ecological discourse. Activists and wildlife biologists took advantage of this opportunity and, through skilful strategic activities oriented at undermining old institutions and creating new ones, managed to induce the government to implement a new policy paradigm. New discourse on the wolf proved a crucial factor for instigating policy transformation. The paper diverged from the dominant way of describing wolf recovery in Europe as a largely impersonal process connected with changing structural and biological parameters. Instead, it highlighted the relevance of bottom-up initiatives from environmental actors for the implementation of conservation institutions and for the subsequent expansion of the species.

## Supporting information

S1 Appendix Interview guide. (PDF)
