# Supplementary material for: What makes a major change of wildlife management policy possible? Institutional analysis of Polish wolf governance
Source: PLoS One. 2020 Apr 23;15(4):e0231601. doi: 10.1371/journal.pone.0231601 (PMC7179832; doi:10.1371/journal.pone.0231601)
Supplement: S1 Appendix — (PDF) [file pone.0231601.s001.pdf]

S1 Appendix. Interview guide

English version

1. Introduction (welcome, thank you, information about the study and asking for a consent to take part in the study and to record the interview)
2. Questions
    1) Since how long have you been interested in wolf issues?
    2) Were you involved into the discussions connected with the protection of the wolf in the 1990s? What was your position? Why? Were you taking any actions to influence the process of listing wolf as a protected species?
    3) Do you remember what contributed to the protection of the wolf in 1998? Which societal groups were supporting this decision and which were opposing it? Why? Which arguments were used to support or oppose wolf protection?
    4) In your opinion, which social actors had the greatest influence on the decision to protect the wolf? What was the role in this process of: (a) wildlife biologists; (b) non-governmental organizations; (c) hunters; (d) foresters; (e) other groups suggested by the interviewee? What made some groups more influential than others? Can you name particular individuals who considerably influenced the decision to protect the wolf in 1998? Which actions each of the group took to support/prevent wolf protection?
    5) In hindsight, how do you assess the decision to protect the wolf?
3. Thank you for participating in the study.

Polish version

1. Wprowadzenie (przywitanie się, podziękowanie za spotkanie, informacja o projekcie badawczym, prośba o zgodę na wywiad i jego nagrywanie).
2. Pytania:
    1) Od jak dawna interesuje się Pani/Pan tematem wilka?
    2) Czy była Pani/Pan zaangażowana w dyskusje wokół wprowadzenia ochrony wilka w połowie lat 90-tych? Jakie było Pani/Pana stanowisko? Dlaczego? Czy podejmowała Pani/Pan jakieś działania, aby wpłynąć na objęcie wilka ochroną w owym czasie?

3) Czy pamięta Pan/Pani jak doszło do objęcia wilka ochroną w 1998 roku? Jakie środowiska popierały tę decyzję, a jaki były jej przeciwne? Dlaczego? Jakie argumenty za i przeciw ochronie wilka były wtedy wysuwane?
4) Jakie podmioty Pani/Pana zdaniem miały największy wpływ na podjęcie tej decyzji? Jaką rolę Pani/Pana zdaniem w tym procesie odegrały: (a) środowiska naukowe związane z badaniami nad wilkami? (b) organizacje społeczne, (c) myśliwi, (d) leśnicy, (e) inne grupy wymienione przez rozmówcę? Na czym polegała siła tych środowisk? Czy może Pani/Pan wymienić konkretne osoby, które miały duży wpływ na podjęcie decyzji o objęciu wilka ochroną w 1998? Jakie działania były podejmowane przez każdą ze stron dyskusji, żeby wprowadzić/zapobiec ochronie wilka?
5) Jak z perspektywy czasu ocenia Pan/Pani decyzję o objęciu wilka ochroną?

3. Podziękowanie za udział w badaniu.
